# Metagenomic Assessment of the Pathogenic Risk of Microorganisms in Sputum of Postoperative Patients With Pulmonary Infection

**DOI:** 10.3389/fcimb.2022.855839

**Published:** 2022-03-03

**Authors:** Junji Chen, Lianjie Sun, Xiaoying Liu, Qixiang Yu, Kaijie Qin, Xuejie Cao, Jianwei Gu

**Affiliations:** ^1^ Department of Cardiovascular Surgery, Ruijin Hospital, Shanghai Jiao Tong University School of Medicine, Shanghai, China; ^2^ Genoxor Medical Science and Technology Inc., Zhejiang, China

**Keywords:** pulmonary infection, sputum microbiome, metagenomic next-generation sequencing, pathogen risk, limit of detection, opportunistic pathogen, strain profiling

## Abstract

Respiratory infections are complicated biological processes associated with an unbalanced microbial community and a wide range of pathogens. To date, robust approaches are still required for distinguishing the pathogenic microorganisms from the colonizing ones in the clinical specimens with complex infection. In this study, we retrospectively analyzed the data of conventional culture testing and metagenomic next-generation sequencing (mNGS) of the sputum samples collected from 50 pulmonary infected patients after cardiac surgery from December 2020 and June 2021 in Ruijin Hospital. Taxonomic classification of the sputum metagenomes showed that the numbers of species belonging to bacteria, fungi, and viruses were 682, 58, and 21, respectively. The full spectrum of microorganisms present in the sputum microbiome covered all the species identified by culture, including 12 bacterial species and two fungal species. Based on species-level microbiome profiling, a reference catalog of microbial abundance detection limits was constructed to assess the pathogenic risks of individual microorganisms in the specimens. The proposed screening procedure detected 64 bacterial pathogens, 10 fungal pathogens, and three viruses. In particular, certain opportunistic pathogenic strains can be distinguished from the colonizing ones in the individual specimens. Strain-level identification and phylogenetic analysis were further performed to decipher molecular epidemiological characteristics of four opportunistic etiologic agents, including *Klebsiella pneumoniae*, *Corynebacterium striatum*, *Staphylococcus aureus*, and *Candida albicans*. Our findings provide a novel metagenomic insight into precision diagnosis for clinically relevant microbes, especially for opportunistic pathogens in the clinical setting.

## Introduction

As is well known, postoperative pulmonary infections are characterized by cough, phlegm, shortness of breath, chest pain, and temperature above 38 degrees (Conde and Lawrence, 2008). The etiological agents causing respiratory tract infections (RTIs) are usually related to a wide variety of pathogenic microbes (Cappelletty, 1998; Chowdhary et al., 2016; Kutter et al., 2018). For instance, a study on 426 patients with suspected lower RTIs has revealed that the most prevalent bacterial species isolated from the specimens (i.e. sputum, endotracheal secretion, and bronchial washing) were *Pseudomonas aeruginosa* and *Haemophilus influenzae* from Gram-negative organisms, and *Streptococcus pneumonia* and *Staphylococcus aureus* from Gram-positive (Khan et al., 2015). Several studies on respiratory infections have reported that *Schizophyllum commune* belonging to filamentous basidiomycetes is an emerging fungal pathogen, and its culture and species identification need a couple of weeks under specific culture media (Chowdhary et al., 2013; Chowdhary et al., 2016). Although traditional culture methods are still the gold standard for clinically microbiological testing, the new omics approaches particularly metagenomics have facilitated culture-free, faster detection for different types of pathogens (e.g. bacteria, viruses, and fungi) in complicated infectious diseases (Deurenberg et al., 2017; Ferone et al., 2020). Besides, many members of the human respiratory tract microbiota are colonizing opportunistic pathogens that are normally harmless and, when the microbial homeostasis becomes disrupted, can cause infections (Price et al., 2017).

Taxonomic profiling *via* metagenomic next-generation sequencing (mNGS) techniques has enabled higher sensitivity and resolution than does conventional culture approaches for better understanding of microbial compositions present in the sputum specimens (Human Microbiome Project Consortium, 2012; Finch et al., 2015; Yan et al., 2016; Ditz et al., 2020; Dicker et al., 2021b). According to 16S rRNA gene amplicon sequencing of sputum samples and clinically measured phenotypes of patients with bronchiectasis, a prospective observational study has revealed that the risk of exacerbation and long-term outcomes are associated with the reduced diversity of sputum microbiome, particularly dominated by bacterial genera *Pseudomonas*, *Enterobacteriaceae*, and *Stenotrophomonas* (Dicker et al., 2021b). Another study has also demonstrated that the dominance of *Proteobacteria* in the sputum microbiome is significantly associated with the neutrophil activation pathway and increased mortality in chronic obstructive pulmonary disease (Dicker et al., 2021a). Besides, Yan et al., have reported that the “near-complete” bacterial genomes could be reconstructed for the highest-abundant pathogens through sputum massive metagenomic sequencing (Yan et al., 2016).

To date, how to distinguish microbial colonization and/or infection is still a vital issue for pathogen identification under hospital settings. To address the above issue, the sputum microbiota of a cohort of postoperative patients with pulmonary infection were investigated based on the data of mNGS and culture testing. Metagenomic analysis was performed to reveal the taxonomic diversity of the sputum microbial community. Based on species-level microbiome profiling, a reference catalog of abundance detection limits was constructed to assess the pathogenic risk of individual microorganisms, which was further compared with the species detected by culture. Subsequently, strain-level identification and phylogenetic analysis were carried out to characterize the clinically relevant pathogens. The scheme of microbial abundance detection limits proposed in this manuscript may provide a useful reference to metagenomic surveillance for opportunistic pathogenic microbes.

## Materials and Methods

### Patients and Samples

This retrospective analysis was performed for the patients with suspected lung infections after cardiac surgery in Ruijin Hospital. In this study, the postoperative patients were enrolled based on abnormal imaging changes represented by chest X-ray, including chest infiltration, consolidation, cavity, and pleural effusion. Pulmonary infection was determined if at least two of the following three options were satisfied: 1) fever greater than 38℃, 2) the number of white blood cells beyond the routine detection limits, 3) occurrence of purulent airway secretion. Sputum specimens of the patients were collected from December 2020 and June 2021 (**Table S1**).

### Specimen Culture

According to the cultivation procedure for bacteria and fungi in the diagnostic laboratory of Ruijin Hospital, the routine isolation media were used, including blood agar, eosin methylene-blue (EMB) agar, chocolate agar, Mueller-Hinton agar, sabouraud dextrose agar, and cooked meat medium (Oxoid, UK). Chocolate agar and EMB agar plates were incubated in 5-10% CO_2_ at 37°C for 48-72 hours; blood agar, Mueller-Hinton agar, and cooked meat medium plates were incubated at 37°C for 24-48 hours. Sabouraud dextrose agar plates suitable for the isolation of *Candida albicans* were incubated at 28°C and 35°C for 24-48 hours, respectively. Species identification of isolated strains was then carried out using the VITEK-2 Compact Instrument (bioMérieux, France) (Melhem et al., 2014).

### Experiments of mNGS

For the preprocessing of specimens, sputum samples were homogenized by the dithiothreitol (DTT) treatment as proposed by Terranova et al. (Terranova et al., 2018). Approximately 200 μL sputum per sample was resuspended by 600 μL of DTT solution (1% concentration) and 20 μL of proteinase K (20 mg mL^-1^), followed by incubation at 56°C for 20 mins. Cells were removed by centrifugation to reduce host-background nucleic acid. Total DNA was extracted using HostZERO Microbial DNA Kit (Zymo Research, USA) following the manufacturer’s instructions. DNA extraction yield was quantified using a Quant-iT dsDNA HS Assay Kit and Qubit 3.0 Fluorometer (Thermo Scientific, USA). Enzymatic shearing was employed for the fragmentation (~200 bp) of DNA molecules and the libraries were then constructed using the Nextera XT DNA Library Preparation Kit (Illumina, USA). A certain number of PCR cycles (5-7) were applied to produce 1 µg of the metagenomic library. The quality of the libraries was assessed by a 2100 Bioanalyzer using the High Sensitivity DNA Assay (Agilent Technologies, USA). Metagenome shotgun sequencing in a single-end 75-bp mode was performed using the NextSeq 500/550 High Output Kit (92 cycles) on an Illumina NextSeq 550 sequencer. The samples as the No-Template Control (NTC) were sequenced simultaneously to assess contaminations during the wet-lab experiments.

### Bioinformatics Analysis of Species-Level Abundance Profiling

Raw sequencing data were first subjected to a quality control process for trimming adapter sequences, removing low-quality tails and reads by Trimmomatic v0.36 (Bolger et al., 2014). Next, the reads mapping to the human reference genome GRCh37 were excluded using the short-read alignment tool Bowtie v2.2.6 (Langmead and Salzberg, 2012). To remove identical duplicated reads considered as technical artifacts by PCR (Martin et al., 2018), de-duplication was performed using an in-house Perl script. Taxonomic classification of microbial reads was conducted using Kraken v2.0.9-beta (Wood et al., 2019) and a custom *k*-mer database constructed using 51,543 genomes of ~27,000 species from the NCBI assembly databases (Kitts et al., 2016). To produce species-level abundance estimates, the number of reads in the Kraken classification report was further estimated by the Bayesian algorithm implemented by Bracken v2.2 (Lu et al., 2017). The microbial taxa bearing putative contamination during the experiments were removed from the final profiles based on the information of NTC samples in each batch: the ratio of read count (sputum sample)/read count (NTC) less than 10 for a specified species. Using a similar way to TPM (transcripts per million) described by Li et al. (Li et al., 2009), the estimates of percentage relative abundance of each species were computed for bacteria, viruses (excluding phages), and fungi, respectively.

To investigate pathogenic signals in the sputum microbiome, we proposed a rule of thumb to evaluate detection limits of pathogenic risks for individual organisms through species-level abundance profiling. Briefly, the values of the mean and standard deviation of relative abundances were calculated according to the estimates of each species across the samples. In this study, limits of detection (LOD) for prompting pathogenic potential of a specified species were estimated through the filtering criteria below: read count > 100; relative abundance > 0.05; presence in at least three samples; the mean plus the standard deviation of the abundance values as the LOD of the pathogenic risk.

### Strain-Level Phylogenetic Analysis

Strain-level profiling analysis was conducted based on single-nucleotide variants (SNVs) using the StrainPhlAn v3.0 package (Truong et al., 2017). Briefly, metagenomic reads per sample were first aligned to the marker gene database of MetaPhlAn v3.0 (Beghini et al., 2021). The sample-specific consensus sequences from the markers were reconstructed for each species’ most abundant strain and the species-specific markers were extracted from MetaPhlAn for identifying the homologous sequences in the reference isolate genomes using blastn (Altschul et al., 1990). Based on sequence alignment, maximum-likelihood phylogenetic trees were reconstructed using RAxML (Stamatakis, 2014) and then visualized using MEGA10 (Kumar et al., 2018). Genome sequences of the isolated strains were retrieved from the NCBI Assembly database and were used for phylogenetic reconstruction together with the strains detected in the metagenomic samples. The program MetaMLST v1.2.2 was employed to predict the known and novel Sequence Type (ST) of the most abundant strains in metagenomic samples (Zolfo et al., 2017). Since it is complicated to resolve the phylogeny of the diploid fungi, an alternative pipeline was used to build the phylogenetic tree according to multilocus sequence typing (MLST) analysis of seven loci (Hirakawa et al., 2015b). Seven MLST allele sequences were reconstructed from the metagenomes using MetaMLST, and the related gene sequences from the isolate genomes were processed using FastMLST (Guerrero-Araya et al., 2021). Multiple sequence alignment was performed with MUSCLE (Edgar, 2004), and the phylogeny was inferred with RAxML.

### Statistical Analysis of Community Diversity

The analysis of *α*-diversity and two sample T-test were conducted using the package fossil v0.4.0 (Chao, 1987) based on the Chao1 index representing community richness. To estimate the community compositional variation of the samples between groups, *β*-diversity was measured by calculating the Bray-Curtis distance matrix using Vegan v2.5-7 (Dixon, 2003). Differences in the rank dissimilarities within and between groups were inferred using the non-parametric ANOSIM test of 1,000 permutations. All statistical analyses and visualizations were performed in R v4.1.0 (R Core Team, 2021).

## Results

### Characteristics of Patients and Samples

In this retrospective study, we analyzed the data of microbiological testing on sputum samples from postoperative patients that were diagnosed with pulmonary infection. During the 7-month study, the specimens were collected from 50 adults aged from 34 to 81 years with a median of 64 years. Approximately three quarters (n = 36; 72%) were male. Participant characteristics are listed in **Table S1**, including body mass index (BMI), dates of both cardiac surgery and specimen sampling, patient outcomes, National Early Warning Score (NEWS) (Smith et al., 2013), and Sequential Organ Failure Assessment (SOFA) scores upon admission (Vincent et al., 1998). The median time of sample collection was day 3 after the cardiac surgery. The samples were then subject to conventional culture and mNGS testing for species identification. DNA mNGS testing yielded an average of 22.9 million reads for each specimen (**Table S2**).

### Species Identification and Pathogenic Risk

As shown in **Table S1**, the culture testing reported 12 bacterial species and two fungal species across all the samples. It resulted in one species detected in each of the 16 sputum samples, two species in five samples, and three species in one sample (**Table 1**). None of the microorganisms was detected in the remaining tested samples (n = 28; 56%) by the culture method. On the contrary, a substantial number of microorganisms were identified by mNGS testing. The numbers of microorganisms assigned to the domains bacteria, fungi, and viruses were 682, 58, 21, respectively. Particularly, the numbers of bacterial and fungal species detected by mNGS testing were much higher than those by culture in the individual samples (**Table 1**). The average number of bacterial species in each sample was 90 with a range from 4 to 219. The average number of fungal species in each sample was 5 with a range from 1 to 17. Besides, the viral species were identified in more than half (n = 31; 62%) of the samples through mNGS; whereas the routine culture approach used in our study can support isolation and identification for bacteria and fungi, but not for viruses.

**Table 1:** Summary of the number of microorganisms detected by culture and mNGS testing for the individual sputum specimens.

ID	Culture testing		mNGS testing					
			Screening by read count			Screening by LOD		
	Bacteria	Fungi	Bacteria	Fungi	Viruses	Bacteria	Fungi	Viruses
S1	1	0	120	3	0	1	0	0
S2	1	1	8	2	1	1	1	0
S3	1	0	34	3	1	3	0	0
S4	0	0	139	3	0	1	1	0
S5	0	0	26	6	0	1	0	0
S6	0	0	132	11	4	4	2	1
S7	1	0	125	11	4	3	1	0
S8	1	0	128	13	3	2	1	1
S9	0	0	173	5	1	2	0	0
S10	1	0	175	3	1	1	0	0
S11	0	0	19	4	8	3	1	0
S12	0	2	7	6	0	1	2	0
S13	0	1	151	17	2	4	1	0
S14	1	0	79	11	4	2	1	0
S15	1	0	176	14	2	1	0	0
S16	1	0	39	1	0	1	0	0
S17	0	0	63	6	1	3	1	0
S18	1	0	77	4	0	4	1	0
S19	0	0	97	5	3	4	1	0
S20	0	0	138	14	1	2	1	0
S21	1	0	219	5	1	4	0	0
S22	0	0	46	4	3	1	0	0
S23	0	0	79	2	0	5	1	0
S24	0	0	207	5	0	3	0	0
S25	1	0	15	5	1	1	1	1
S26	0	0	25	3	1	3	0	0
S27	0	0	141	5	6	1	1	1
S28	0	0	192	3	1	4	1	0
S29	2	1	42	5	3	1	1	1
S30	0	0	42	2	0	1	0	0
S31	0	0	43	6	1	3	1	0
S32	2	0	197	3	1	2	0	0
S33	0	0	47	4	0	4	0	0
S34	0	0	85	2	0	4	0	0
S35	0	0	98	2	1	5	0	0
S36	1	0	4	2	0	2	0	0
S37	2	0	4	5	0	1	1	0
S38	1	0	216	4	0	2	0	0
S39	0	0	196	13	0	3	1	0
S40	1	0	15	6	1	1	0	0
S41	1	0	46	2	2	2	1	0
S42	0	0	101	2	1	7	0	1
S43	0	0	163	3	1	7	1	0
S44	1	1	50	4	3	2	2	1
S45	0	0	125	2	1	2	0	1
S46	0	0	103	2	0	5	0	0
S47	0	0	22	6	1	0	1	0
S48	0	0	24	3	0	1	0	0
S49	0	0	42	4	0	0	0	0
S50	0	0	14	3	0	1	0	0

To assess the pathogenic risk of individual microorganisms, we proposed the abundance LOD for screening candidate pathogens based on mNGS assays and species-level abundance profiles of bacteria, fungi, and viruses, respectively (**Table S3**). Most of the bacterial and fungal species detected by the culture approach were present in the list of the pathogens that were screened out in the individual samples (**Table 2**; the full list shown in **Table S1**). For instance, both culture and mNGS testing for patient #2 identified *B. cenocepacia* and *C. albicans*, which were predominant pathogens in the bacterial (86.3% abundance) and fungal community (98.7%), respectively. For patient #44, the mNGS approach identified five pathogens *C. striatum* (56.2%), *S. aureus* (39.2%), *C. albicans* (93.1%), *A. flavus* (5.5%), and Human alphaherpesvirus 1 (99.8%). Of these, *S. aureus* and *C. albicans* were also detected by culture. According to the LOD (30.8%) of *K. pneumoniae*, this bacterium was assessed as a pathogen in the four patients: 85.4% for #1, 87.1% for #4, 97.7% for #16, and 72.1% for #40. The sputum culture was positive for *K. pneumoniae* in these patients except for #4. The mNGS approach for patient #4 identified *K. pneumoniae* (87.1%) and *C. albicans* (97.8%) as pathogens; whereas, none of them was detected by culture. *C. albicans*, an opportunistic pathogenic yeast, was the most prevalent fungal pathogen detected in the sputum metagenomes of 15 patients (**Table S1**). In addition, human alphaherpesvirus 1 was the most frequently detected pathogen that was identified in six patients. Generally, according to our proposed mNGS screening criteria, the predicted microbial targets bearing pathogenic risk not only well matched the ones detected by culture, but also provided additional and prioritized pathogens missed by culture. It should provide complementary evidence for microbiological diagnosis in the clinical setting.

**Table 2:** List of selected sputum specimens from post-surgery infected patients tested in this study.

ID	Patient’s sex, age^a^	Culture testing^b^	Pathogens based on mNGS testing with LOD		
			Bacteria	Fungi	Viruses
1	m, 71	*Klebsiella pneumoniae*	*Klebsiella pneumoniae* (85.4%);	–	–
2	m, 67	*Burkholderia cenocepacia*; *Candida albicans*	*Burkholderia cenocepacia* (86.3%);	*Candida albicans* (98.7%);	–
3	f, 77	*Enterobacter cloacae* complex	*Staphylococcus epidermidis* (51.7%); *Enterobacter hormaechei* (23.8%); *Enterococcus faecalis* (5.7%);	–	–
4	m, 49	–	*Klebsiella pneumoniae* (87.1%);	*Candida albicans* (97.8%);	–
7	m, 65	*Stenotrophomonas maltophilia*	*Stenotrophomonas maltophilia* (50.9%); *Mogibacterium timidum* (8.0%); *Enterococcus faecalis* (7.9%);	*Talaromyces marneffei* (31.3%);	–
10	m, 65	*Serratia marcescens*	*Serratia marcescens* (56.8%);	–	–
11	m, 79	–	*Abiotrophia defectiva* (37.0%); *Staphylococcus epidermidis* (30.4%); *Klebsiella oxytoca* (5.1%);	*Candida albicans* (89.8%);	–
14	m, 79	*Acinetobacter baumannii*	*Stenotrophomonas maltophilia* (57.0%); *Acinetobacter baumannii* (21.0%);	*Candida parapsilosis* (98.5%);	–
16	f, 71	*Klebsiella pneumoniae*	*Klebsiella pneumoniae* (97.7%);	–	–
25	m, 46	*Acinetobacter baumannii*	*Acinetobacter baumannii* (99.2%);	*Candida albicans* (99.7%);	Human betaherpesvirus 5 (99.6%);
26	m, 64	–	*Parvimonas micra* (51.7%); *Streptococcus anginosus* (13.1%); *Streptococcus milleri* (13.0%);	–	–
27	m, 70	–	*Corynebacterium striatum* (92.9%);	*Candida albicans* (99.0%);	Human alphaherpesvirus 1 (53.6%);
28	f, 55	–	*Parvimonas micra* (27.9%); *Streptococcus oralis* (23.1%); *Atopobium rimae* (6.6%); *Atopobium parvulum* (5.1%);	*Candida albicans* (89.9%);	–
36	m, 71	*Acinetobacter baumannii*	*Acinetobacter baumannii* (50.8%); *Staphylococcus aureus* (48.9%);	–	–
40	m, 62	*Klebsiella pneumoniae*	*Klebsiella pneumoniae* (72.1%);	–	–
44	f, 66	*Staphylococcus aureus*, *Candida albicans*	*Corynebacterium striatum* (56.2%); *Staphylococcus aureus* (39.2%);	*Candida albicans* (93.1%); *Aspergillus flavus* (5.5%);	Human alphaherpesvirus 1 (99.8%);

a 'f' for female and 'm' for male.

b ‘-’ denotes none of the organisms is detected.

### Community Structure of the Sputum Microbiota

**Figure 1:**
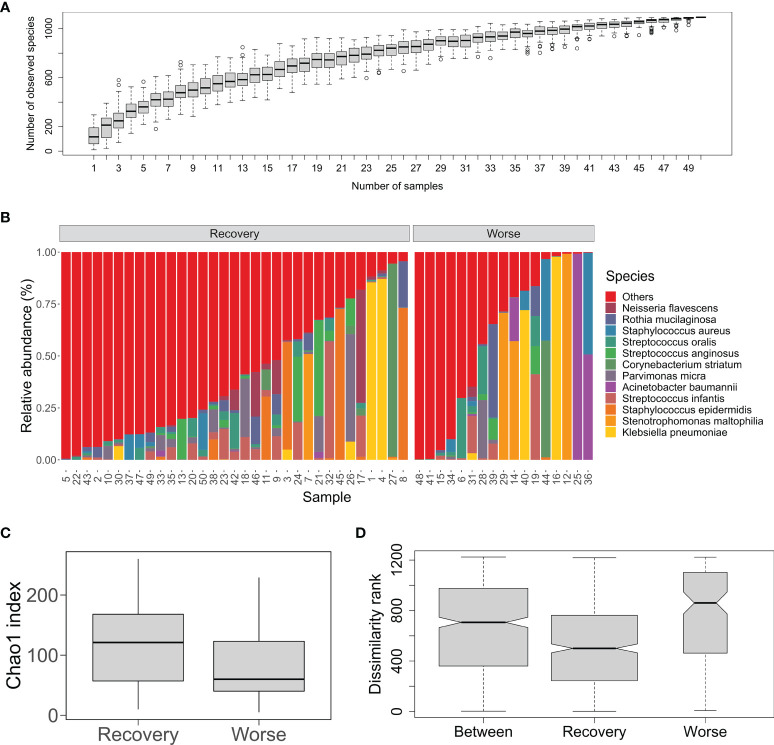
Bacterial community structure and diversity of the sputum microbiota from the post-surgery patients with pulmonary infection. **(A)** An accumulation curve of the microorganisms in the sputum samples. The boxplot denotes the species richness according to the observed species numbers for a given number of samples collected from 100 random permutations of all the samples. **(B)** Percent stacked barplot for species-level taxonomic profiling of the sputum bacterial microbial communities. Labels stand for the top 12 abundant species. The samples are grouped by clinical outcomes Recover and Worse. **(C)** Chao1 index of the sputum microbiota from the two groups Recover and Worse. **(D)** The ANOSIM boxplot ranks between and within the Recover and Worse groups.

To explore organismal composition in the sputum microbiota, the mNGS dataset containing all sequenced metagenomes was analyzed. We focused on the diversity of bacterial communities bearing high species richness compared to the fungal and viral communities (**Table 1**). The species accumulation curve tended to be smooth, indicating that the number of bacterial species would vary slowly with the increase in sample size (**Figure 1A**). The profile of percentage taxonomic abundances across the sputum metagenomes was displayed in **Figure 1B**. According to the average values of abundances, the top five abundant species were *K. pneumonia* (7.3%), *S. maltophilia* (5.6%), *S. epidermidis* (4.9%), *S. infantis* (4.8%), and *A. baumannii* (3.6%) (**Figure 1B**). It was observed that certain species that were remarkably dominating in the community of the individual samples were estimated to be etiologic agents mentioned above, such as *K. pneumonia* in #1/#4/#16/#40, *A. baumannii* in #25/#36, *N. flavescens* in #9/#17/#46, and *S. anginosus* in #13/#19/#21/#24/#26 (**Table S1**). Next, we compared the community structural dynamics of the sputum microbiota between two clinical outcomes: Recovery and Worse. The α-diversity analysis of community richness indicated that no significant difference (*p* = 0.125) was found between the two groups (**Figure 1C**). Based on the *β*-diversity ANOSIM test (R = 0.173, *p* = 0.008), it was apparent that the mean of ranked dissimilarities between groups was lower than that within the group Worse (**Figure 1D**). Meanwhile, the low R-value suggested a weak effect of clinical outcomes on the microbial communities. Both *α*- and *β*-diversity analyses indicated that the bacterial community diversity was less associated with clinical outcomes of postoperative infected patients, probably due to diverse pathogens dominating the individual samples.

### Strain-Level Characteristics of Pathogens

**Figure 2:**
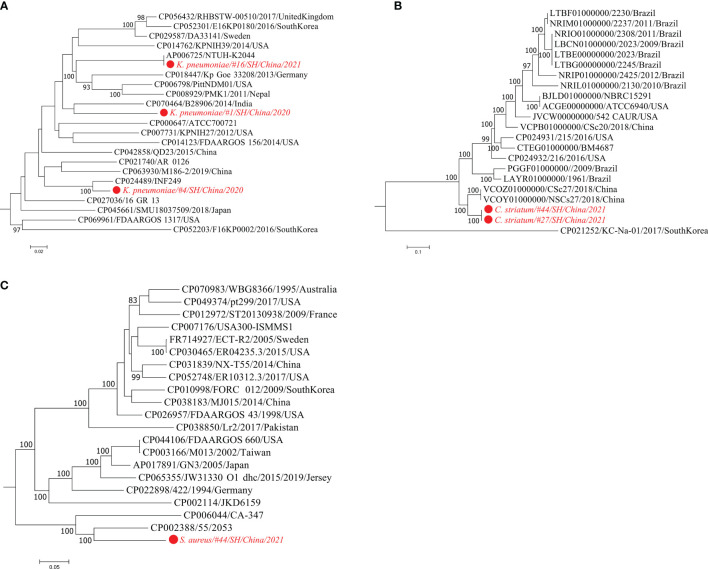
Maximum-likelihood phylogenetic trees of three bacterial pathogens. Using StrainPhlAn trees are built based on the sample-specific consensus sequences of marker genes from *K. pneumoniae* **(A)**, *C. striatum* **(B)**, and *S. aureus* **(C)**, respectively. The strains detected in the metagenomic samples are labeled in red. The isolate genomes are labeled by the GenBank accessions, strain identifiers, isolation country, and year. Bootstrap support values greater than 80% are displayed.

Strain identification is of significant importance for better understanding microbial pathogenicity and transmission of clinically relevant microorganisms (Nayfach et al., 2016). According to the pathogen list assessed by mNGS testing (**Table S1**), strain-level profile analysis was performed for four opportunistic pathogenic species *K. pneumoniae*, *C. striatum*, *S. aureus*, and *C. albicans*. StrainPhlAn analysis displays strain-level phylogenetic trees visualizing the relatedness between the metagenome-recovered strains and clinical bacterial isolates with publicly genome sequences (**Figure 2**). *K. pneumoniae* strains were reconstructed from the metagenomic samples of three patients #1/#4/#16 (**Figure 2A**). The analysis of metaMLST showed that the strain from patient #4 was *K. pneumoniae* of ST29 and the other two strains were represented by novel STs. Consistently, a *K. pneumoniae* strain derived from #4 was most closely related to a multidrug-resistant ST29 strain (CP024489/INF249) isolated from urinary tract infection (Gorrie et al., 2018) (**Figure 2A**). The strain from #16 was closely related to the *K. pneumoniae* strain NTUH-K2044 (AP006725) causing liver abscess and meningitis (Wu et al., 2009). In addition, two *C. striatum* strains from #27/#44 were phylogenetically placed together (**Figure 2B**), and their closest relatives were the two strains (VCOZ00000000 and VCOY00000000) of *C. striatum* isolated from influenza patients with secondary lower respiratory tract bacterial infections (Qin et al., 2020). Besides, the *S. aureus* strain from #44 was neighboring to the strain 55/2053, which caused the global pandemic of Panton-Valentine leukocidin-producing *S. aureus* in Europe and the United States in the 1950s (Zuo et al., 2021) (**Figure 2C**).

**Figure 3:**
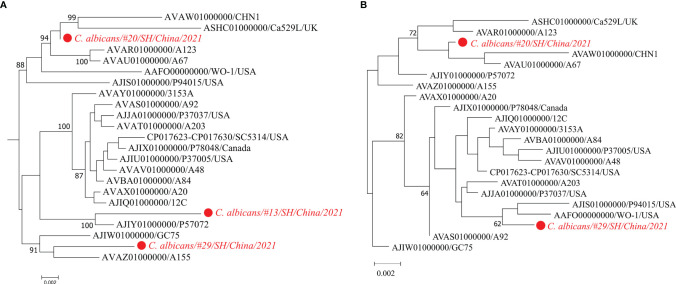
Maximum-likelihood phylogenetic trees of the fungal pathogen *C. albicans*. Trees are built according to the StrainPhlAn analysis **(A)** and MLST analysis **(B)**, respectively. The strain of *C. albicans* in the metagenome sample #13 is excluded from the MLST tree due to its low sequencing depth and failure to reconstruct the MLST loci-sequences.

**Figure 3** shows the phylogeny of three metagenomic strains of *C. albicans* from #13/#20/#29 together with the isolated strains of *C. albicans*, which is a well-known human fungal pathogen responsible for painful mucosal infections (Kim and Sudbery, 2011; Gulati and Nobile, 2016). Phylogenetic relationships of these fungal strains were investigated by the StrainPhlAn-based tree and the MLST-based tree, respectively. Both trees show that the *C. albicans* strain from #20 was closely related to the strains CHN1, Ca529L, A123, and A67.

## Discussion

Rapid species identification followed by assessment of microbial colonization and pathogenesis associated with infections is always a significant issue for public health (Stressmann et al., 2017; Martin and Bachman, 2018). In recent years, culture-free high-throughput sequencing methods have successively uncovered many microbial colonizers in the nasopharynx, oropharynx, airway, trachea, and lung of healthy humans, such as *Streptococcus*, *Haemophilus*, *Corynebacterium*, *Staphylococcus*, *Klebsiella*, et al. (Charlson et al., 2011; Martin and Bachman, 2018; Paling et al., 2020). Microbiota balance of specific body sites, including the respiratory tract, is thought to be closely related to host health and disease (Man et al., 2017). Disruption of the microbiota homeostasis underlying the state of diseases enables changing the proportions of certain opportunistic pathogenic strains, thereby facilitating the proliferation of a complex community dominated by these strains and further causing infectious diseases (Haldar et al., 2020; Dicker et al., 2021b). Inference on the risk of colonization and infection by opportunistic pathogens has important implications for the diagnostics of these clinically relevant strains. Thus, we explored the screening procedure of mNGS-based species abundance profiles from the patients with pulmonary infection, further proposing LOD to assess the pathogenic risk in the sputum microbiome in this manuscript.

Microbiome-wide estimation on pathogenic risk for diverse microorganisms is beneficial for distinguishing the pathogenic strains from the colonizing strains in the clinical specimens. In this retrospective analysis, the final pathogen list generated by mNGS-based screening procedures encompassed nearly all the species identified by culture and some additional pathogens associated with infection. Of these pathogens detected in the sputum microbiome, many are human colonizers and also opportunistic etiologic agents, e.g. *K. pneumoniae* (Chung, 2016), *S. marcescens* (Khanna et al., 2013), *S. oralis* (Adams et al., 2017), *S. mitis* (Adams et al., 2017), *C. striatum* (Alibi et al., 2017), and *S. aureus* (Feng et al., 2008). Gram-positive bacterium *S. pneumoniae* is one of the prevalent commensal residents of the nasopharynx and also an opportunistic pathogen that can lead to pneumonia, bacteremia, meningitis, and otitis media (Shak et al., 2013). Herein, *S. pneumoniae* was present in more than half (n = 28) of the samples (**Table S3**). Based on the LOD of 3.6% for this bacterium, it was identified as a pathogen in the three patients: 15.7% abundance in #6; 7.4% in #33; 8.7% in #42. *S. marcescens*, a Gram-negative opportunistic nosocomial pathogen, is commonly involved in catheter-associated bacteremia, urinary tract infections, and wound infections of hospitalized patients (Khanna et al., 2013). *S. marcescens* was detected as a singular pathogen (56.8%) in #10, which was consistent with species identification by culture. *C. albicans* (36.3%) is normally a harmless commensal yeast and also a prevalent opportunistic pathogen that can cause symptomatic infections of mucosal membranes (Kim and Sudbery, 2011). This common fungus was present in about half (n = 23) of the samples (**Table S3**), and it was further assessed as a high-risk pathogen dominating the fungal community of 30% of the samples. These results suggest that the proposed LOD value of pathogenic risk may be a promising clinical indicator for infections caused by opportunistic etiological agents. However, it still needs a bigger sample group to provide robust references of microbial abundances for statistical estimation on LOD of pathogenic risk.

On the other hand, mining metagenomic data can provide more accurate taxonomic classification even at the single-strain resolution, which can be further used to interpret microbial pathogenesis, antimicrobial resistance, and transmission of the newly detected strain (Nayfach et al., 2016; Truong et al., 2017). For example, the sputum culture was negative for *K. pneumoniae* in patient #4, whereas *K. pneumoniae* was identified in the corresponding specimen by mNGS. Interestingly, strain profiling of the sputum metagenome revealed that this highly abundant *K. pneumoniae* strain was phylogenetically related to the multidrug-resistant and virulent isolate INF249 belonging to *K. pneumoniae* of ST29 and KL30 (Gorrie et al., 2018) (**Figure 2A**). Additionally, the *K. pneumoniae* strain detected in patient #16 was related to NTUH-K2044, a hypervirulent strain bearing the K1 capsule and ST23 (Wu et al., 2009). Besides, for the fungal typing of *C. albicans*, we identified three strains that were distantly related to each other in the phylogenetic tree (**Figure 2D**). The strain of *C. albicans* isolated from patient #13 was most closely related to *C. albicans* strain P57072, which is isolated from a bloodstream infection (Hirakawa et al., 2015). Since opportunistic etiological agents normally include both disease-causing and commensal strains (Paczosa and Mecsas, 2016; Braz et al., 2020), strain-level phylogeny is an alternative to providing accurate detection of whether it is a pathogenic strain or not.

In summary, we utilized mNGS-based species abundance profiling data to propose a practical screening strategy for the assessment of microbial pathogenic risk in sputum of pulmonary infected patients. The strategy enables reducing the disturbance from colonizers in a microbial community; meanwhile, it could also distinguish pathogenic strains from the colonizing strains in specific body sites. Our work provides a novel metagenomic insight into precision diagnosis for clinically relevant microbes, especially for opportunistic pathogens in the clinical setting.

## Data Availability Statement

The datasets presented in this study can be found in online repositories. The names of the repository and accession number can be found below: https://www.ncbi.nlm.nih.gov/, PRJNA765792.

## Ethics Statement

The studies involving human participants were reviewed and approved by The Ethics Committee of Ruijin Hospital, Shanghai Jiaotong University School of Medicine Review Board (2021-69). The patients/participants provided their written informed consent to participate in this study.

## Author Contributions

Conceived and designed the study: JC and JG. Performed the experiments: JC, XL, and QY. Analyzed the data: XC, LS, and KQ. Wrote and revised the manuscript: JC, LS, and JG. All authors contributed to the article and approved the submitted version.

## Funding

This work was supported by the National Natural Science Foundation of China (81671832).

## Conflict of Interest

The authors LS and XC are employed by Genoxor Medical Science and Technology Inc.

The remaining authors declare that the research was conducted in the absence of any commercial or financial relationships that could be construed as a potential conflict of interest.

## Publisher’s Note

All claims expressed in this article are solely those of the authors and do not necessarily represent those of their affiliated organizations, or those of the publisher, the editors and the reviewers. Any product that may be evaluated in this article, or claim that may be made by its manufacturer, is not guaranteed or endorsed by the publisher.
