# Supplementary material for: Metagenomic Assessment of the Pathogenic Risk of Microorganisms in Sputum of Postoperative Patients With Pulmonary Infection
Source: Front Cell Infect Microbiol. 2022 Mar 3;12:855839. doi: 10.3389/fcimb.2022.855839 (PMC8928749; doi:10.3389/fcimb.2022.855839)
Supplement: Supplementary file 5 [file Table_5.docx]

Supplementary Material

**Table S1.** Summary of clinical traits, the microorganisms detected by culture and VITEK-2, and the pathogens assessed by mNGS and the screening procedure via LOD proposed in this study.

**Table S2.** Statistics of metagenomic sequencing and quality control.

**Table S3**. Species-level relative abundance profiling of microbial community and the LOD values for assessing pathogenic risk of the microorganisms (bacteria, fungi, and viruses) present in the sputum microbiome.

**Table S4**. Species-level read count profiling of microbial community and the LOD values for assessing pathogenic risk of the microorganisms (bacteria, fungi, and viruses) present in the sputum microbiome.
